# Comparative efficacy of six contemporary large language models in prosthodontic patient education: a multi-parameter blinded assessment

**DOI:** 10.1186/s12903-026-08705-9

**Published:** 2026-05-27

**Authors:** Ahmet Doğan Işık, Kaan Yerliyurt

**Affiliations:** Department of Prosthodontics, Faculty of Dentistry, Tokat Gaziosmanpaşa University, Tokat, Turkey

**Keywords:** Artificial intelligence, Large language models, Prosthodontics, Patient education, Chatbot

## Abstract

**Background:**

This study aimed to evaluate and benchmark the proficiency of six major large language models (LLMs)—specifically the versions publicly available as of March 2025 (ChatGPT-4o, Claude 3.7 Sonnet, Microsoft Copilot, DeepSeek-V2, Gemini 2.0, and Grok-3)—in addressing prosthodontic patient inquiries across various clinical dimensions, including scientific accuracy, comprehensiveness, clarity, and relevance.

**Methods:**

In this descriptive, cross-sectional comparative study, ten standardized patient questions encompassing key prosthodontic topics (fixed prostheses, removable prostheses, dental implants, and aesthetic restorations) were systematically posed to six current LLM platforms. Each model was assigned a prosthodontic specialist persona through standardized prompts. A total of 60 independent responses (representing unique combinations of the 10 questions and 6 models) were obtained. These were evaluated by two experienced prosthodontic specialists (one Professor with 15 + years, one Associate Professor with 8 + years of clinical and academic experience) using a validated 5-point Likert scale. The evaluation was strictly double-blinded; assessors were completely unaware of both the model identities and each other’s ratings. Statistical analyses included Intraclass Correlation Coefficient (ICC) for inter-rater reliability, Cronbach’s alpha for internal consistency, and Kruskal-Wallis H test for performance comparison, with significance set at *p* < 0.05.

**Results:**

Claude 3.7 Sonnet achieved the highest overall mean score (3.79 ± 0.74), followed by Gemini 2.0 (3.75 ± 0.58) and Grok-3 (3.70 ± 0.54). ChatGPT-4o, DeepSeek-V2, and Microsoft Copilot demonstrated varying performance levels (3.40 ± 0.64, 3.68 ± 0.46, and 3.29 ± 0.53, respectively). While no statistically significant differences were observed among models for scientific accuracy (*p* = 0.320), clarity (*p* = 0.184), or relevance (*p* = 0.608), comprehensiveness showed significant variation (*p* = 0.036). Gemini 2.0 provided significantly more comprehensive responses (3.90 ± 0.66) compared to Microsoft Copilot (2.95 ± 0.55). Inter-rater reliability was good (ICC = 0.709, 95% Confidence Interval [CI]: 0.550–0.846, *p* < 0.001), as was internal consistency (Cronbach’s α = 0.709). Critically, none of the evaluated models achieved ratings in the “very good” category (≥ 4.5) across all parameters.

**Conclusion:**

Contemporary LLMs demonstrate moderate-to-good proficiency in addressing prosthodontic patient education queries, with Claude 3.7 Sonnet and Gemini 2.0 currently offering the most balanced performance profiles. While scientific accuracy is comparable across platforms, significant variations exist in information comprehensiveness. Hallucinations remain an inherent risk across all models, and the absence of “very good” ratings indicates substantial limitations that necessitate professional oversight. Future assessments should include qualitative error analysis and layman evaluations to better capture patient perspectives.

**Supplementary Information:**

The online version contains supplementary material available at 10.1186/s12903-026-08705-9.

## Background

The digital transformation of healthcare has accelerated dramatically over the past decade, with artificial intelligence (AI) emerging as a potentially transformative technology across multiple clinical domains [1, 2]. As part of this digital evolution, AI-based chatbot systems have increasingly been integrated into healthcare to support clinical decision-making, patient communication, and health information dissemination. The most significant recent advance in this area is the development of Large Language Models (LLMs). LLMs are highly advanced natural language processing architectures trained on billions of data points, enabling them to understand context, generate human-like text, and engage in logical dialogue without explicit programming [3, 4]. Platforms built on these models, such as ChatGPT, Claude, and Gemini, have demonstrated remarkable capabilities in synthesizing complex information and generating contextually appropriate responses across diverse subject areas [5, 6].

In dentistry, AI integration has progressed from diagnostic imaging analysis and treatment planning optimization to patient education and clinical information dissemination [7–9]. Prosthodontics, as a specialty focused on the restoration and replacement of teeth through fixed prostheses, removable prostheses, and implant-supported reconstructions, demands both precise technical knowledge and effective patient communication [10]. Educating patients in this field presents unique challenges due to the mechanical complexity of the restorations, the wide range of treatment alternatives (from conventional dentures to implant-supported rehabilitations), critical aesthetic considerations, and often substantial financial implications. The complexity of prosthetic treatment options—ranging from single-unit restorations to complete oral rehabilitation—necessitates comprehensive patient education to facilitate informed decision-making and realistic expectation management [11].

Effective patient education in prosthodontics is paramount for treatment success, as it directly influences patient satisfaction, prosthesis adaptation, and long-term maintenance [12]. However, achieving optimal communication is frequently complicated by varying levels of patient health literacy, which significantly impacts the comprehension of complex biomechanical and clinical instructions [13]. Traditional patient education methods, while valuable, face inherent limitations in terms of accessibility, consistency, standardization, and scalability [14]. Patients increasingly seek health information through digital channels, with internet-based resources becoming primary sources of medical knowledge prior to clinical consultations [15, 16]. LLM-based chatbots present a potentially valuable opportunity to address these constraints by providing instantaneous, standardized, and accessible responses to patient inquiries [14, 17].

Recent literature has begun exploring LLM performance in various dental specialties, examining their accuracy in answering examination questions and providing patient information [17–19]. However, several significant knowledge gaps persist. Specifically, this study is designed to explicitly address three critical gaps in the current literature. First, most existing studies have focused predominantly on ChatGPT alone or on earlier model versions, neglecting comprehensive comparisons of multiple contemporary LLM platforms. Second, many investigations have employed single-parameter assessment methodologies—typically focusing solely on accuracy—while overlooking other clinically essential dimensions such as comprehensiveness, clarity, and practical relevance. Third, there remains a distinct lack of specialty-specific benchmarking in prosthodontics, a domain that presents unique patient education challenges due to complex mechanical, biological, and aesthetic considerations. The rapid evolution of LLM technology necessitates continuous reassessment to fill these gaps, as model capabilities can change substantially within months [18, 19].

The evaluation of AI-generated medical information requires multidimensional assessment frameworks that extend beyond simple accuracy measurement. To determine practical clinical utility, four critical parameters must be explicitly defined from the outset: (1) scientific accuracy (the correctness of medical facts and absence of misleading statements), (2) comprehensiveness (the depth, completeness, and inclusion of relevant clinical context), (3) clarity (the accessibility of explanations and appropriate use of lay terminology), and (4) relevance (the direct applicability of the response to the specific patient query). These parameters represent equally critical factors influencing practical clinical utility. Additionally, methodological rigor demands robust assessment of inter-rater reliability and measurement tool validity to ensure reproducibility and credibility of findings [20].

This study addresses these existing knowledge gaps by conducting a systematic comparative evaluation of six prominent, currently available LLM platforms specifically within the context of prosthodontic patient education. Our investigation employs a validated multi-parameter assessment framework, implemented by experienced prosthodontic specialists using a rigorous double-blind methodology. The research aims to provide evidence-based guidance regarding the potential roles, relative strengths, and critical limitations of LLM chatbots in dental patient care contexts. The null hypothesis (*H*_*0*_) of this study posited that there would be no statistically significant differences in performance among the evaluated LLM platforms regarding scientific accuracy, comprehensiveness, clarity, and relevance.

## Methods

### Study design and ethical considerations

This descriptive, cross-sectional comparative study was designed to evaluate LLM chatbot performance in providing prosthodontic patient education responses. As the study involved only publicly available AI chatbot platforms without human participant involvement or personal data collection, formal institutional ethical approval was not required. The study design and reporting adhered to STROBE (Strengthening the Reporting of Observational Studies in Epidemiology) guidelines for observational research [21].

### Selection of large language models

Six contemporary LLM chatbots were selected based on the following criteria: (1) public availability as of March 2025, (2) documented active use in healthcare contexts, (3) representation of different development organizations and architectural approaches, and (4) established natural language processing capabilities. To ensure rigorous reproducibility, all models were accessed and queried during a strictly defined timeframe (March 9–10, 2025). Furthermore, the specific access methods were standardized; all evaluations were conducted using the freely available public tiers (Free Tier / Free Web Access) to reflect the most common real-world usage scenario for patients seeking health information. The selected platforms, their developers, release dates, and architectural characteristics evaluated in this study are detailed in Table 1.

**Table 1:** Characteristics of the evaluated large language models (as of March 9–10, 2025)

Model	Developer	Release / Version Date	Parameter Count	Access Method (Tier)
ChatGPT-4o	OpenAI	May 2024	Undisclosed	Free Tier
Claude 3.7 Sonnet	Anthropic	February 2025	Undisclosed	Free Tier
Microsoft Copilot	Microsoft	Late 2023 / Updated 2024	Undisclosed	Free Web Access
DeepSeek-V2	DeepSeek AI	May 2024	236 Billion (MoE)*	Free Web Access
Gemini 2.0	Google AI	December 2024	Undisclosed	Free Tier
Grok-3	xAI	February 2025	Undisclosed	Free Tier

*MoE Mixture-of-Experts architecture

### Development of patient question set

Ten standardized patient questions were systematically developed to represent commonly encountered inquiries in prosthodontic clinical practice. To strictly reflect the primary clinical context and natural language patterns of the target patient population in Turkey, all interactions with the models (prompts) were exclusively conducted in Turkish, and all LLM responses were generated and evaluated in Turkish. The initial question set underwent a content validation review by three independent prosthodontic specialists (one Professor, two Associate Professors) to ensure clinical relevance, representativeness, and clarity. Based on their feedback, minor syntactic adjustments were made to ensure the queries perfectly mimicked everyday patient vernacular rather than academic terminology. For international readership and reproducibility, the validated Turkish questions were translated into English by two bilingual prosthodontic researchers, ensuring conceptual and semantic equivalence. Both the original Turkish questions and their validated English translations are provided together in Additional File 1. The question set encompassed key prosthodontic topics including fixed prostheses (dental crowns, bridges), removable prostheses (complete and partial dentures), dental implants, and occlusal habits (bruxism). While traditional a priori sample size calculations are typically designed for biological variability in human subjects, the sample size for this digital benchmarking study (10 standardized queries across 6 models, *N* = 60) was determined based on established methodological precedents in high-impact dental AI literature [22]. The statistical robustness of this sample size was verified through two distinct metrics. First, the 10-prompt framework was sufficient to achieve highly significant inter-rater reliability, as evidenced by an overall ICC of 0.709 (*p* < 0.001). Second, a post-hoc power analysis using G*Power (v3.1.9.7) for the ‘Comprehensiveness’ parameter (*p* = 0.036) revealed a large effect size (Cohen’s f = 0.48) and a statistical power (1-ß) of 87%. This exceeds the standard 80% threshold, confirming that the study was adequately powered to detect significant performance differences among the evaluated models.

### Data collection protocol

Data collection was conducted during a strictly defined timeframe (March 9–10, 2025). To ensure rigorous methodological consistency and control for potential confounding variables inherent to LLM interactions, a standardized protocol was implemented:

- Role Assignment: Each LLM was assigned a standardized “prosthodontic specialist” persona to contextualize responses appropriately. The exact prompt administered to all models (in Turkish) was: *“*Sen deneyimli bir protetik diş tedavisi uzmanısın. Lütfen aşağıdaki hasta sorusunu, halktan birinin anlayabileceği açık ve erişilebilir bir dil kullanarak, ancak katı bilimsel doğruluğu koruyarak kapsamlı bir şekilde yanıtla.” (English translation: “You are an experienced prosthodontic specialist. Please answer the following patient inquiry comprehensively, using clear and accessible language suitable for a layperson, while maintaining strict scientific accuracy.“).
- Contextual Isolation: To manage the variable of conversation memory, each question was posed in a completely new, isolated session to prevent contextual influence from previous interactions.
- Parameter Control: By exclusively utilizing the public free tiers, internal model variables (such as sampling temperature) were standardized according to the developers’ default settings, eliminating manual manipulation bias.
- Response Capture and Blinding: Complete chatbot responses were captured verbatim, coded numerically, and randomized to ensure evaluators remained completely blinded to model identity.
- Confounding Variable Management: By implementing the strict isolation, parameter standardization, and blinding procedures detailed above, external algorithmic and evaluator biases were systematically mitigated.

This methodology ensured that each of the 60 responses (6 models × 10 questions) represented an independent data point unaffected by conversation history or evaluator bias.

### Evaluator selection and training

Two independent evaluators, both experienced prosthodontic specialists, participated in the assessment:

- Evaluator 1: Professor with 15 + years of prosthodontic practice and academic experience.
- Evaluator 2: Associate Professor with 8 + years of prosthodontic practice and academic experience.

Prior to evaluation, both assessors underwent structured training regarding the rating criteria and scale interpretation to ensure consistent application of assessment parameters. Evaluators remained completely blinded to both the model identities and each other’s independent ratings throughout the entire evaluation process.

### Evaluation criteria and rating scale

The evaluation framework of the present study was specifically designed in accordance with established contemporary methodologies for assessing AI-generated medical information, which emphasize multi-parameter scoring systems encompassing accuracy, comprehensiveness, and clinical safety. Furthermore, this precise four-parameter framework (assessing comprehensiveness, scientific accuracy, clarity, and relevance) has been successfully validated in recent high-impact dental literature evaluating LLM performance in periodontology [22]. Accordingly, responses were evaluated across four clinically relevant parameters (Scientific Accuracy, Comprehensiveness, Clarity, and Relevance) using a rigorously defined 5-point Likert scale. To ensure standardized and objective grading between the two independent assessors, explicit anchor descriptions were established for each point on the scale (1: Very Poor to 5: Very Good), clearly defining the qualitative distinctions between score levels. For instance, regarding the evaluation of a loose crown inquiry, a ‘Very Good’ (5) response would flawlessly identify potential etiologies (e.g., cement failure or secondary caries), delineate the clinical risks of non-intervention (e.g., aspiration or further dental damage), and provide precise professional guidance. Conversely, a ‘Very Poor’ (1) response is characterized by fundamentally flawed or dangerous advice, such as suggesting the use of household adhesives for re-cementation, thereby posing severe biological and safety risks to the patient. A comprehensive breakdown of the specific anchor descriptions used for all evaluation parameters is detailed in Table 2.

**Table 2:** Anchor descriptions for the 5-point Likert scale evaluation parameters

Score	Scientific Accuracy	Comprehensiveness	Clarity	Relevance
5 (Very Good)	Completely accurate, evidence-based, and contains absolutely no errors.	Covers all critical details, treatment alternatives, and essential patient instructions.	Exceptionally fluent, simple, and easily understood by a layperson. Complex terms are fully explained.	Directly addresses the query with practical, safe, and actionable advice; clearly recommends consulting a dentist.
4 (Good)	Largely accurate, but may contain minor or clinically insignificant omissions.	Covers the main outlines well, but omits some secondary or minor details.	Generally clear, but may contain occasional mild medical jargon or slightly complex phrasing.	Highly relevant and provides useful advice, including a general recommendation to see a dentist.
3 (Moderate)	Generally correct but lacks important updates or contains non-critical flaws.	Only basic aspects are addressed; significant details or alternatives are overlooked.	Requires effort from the patient to comprehend; contains confusing expressions.	Associated with the query but remains generic; actionable advice is weak.
2 (Poor)	Contains significant scientific errors or misleading medical information.	Very superficially addressed; fails to meet even the basic components of the query.	Complex, dense with medical jargon, and very difficult for a patient to understand.	Only indirectly related to the topic; clinical guidance is unclear or absent.
1 (Very Poor)	Contains dangerously inaccurate information that could pose potential health risks.	Contains almost no meaningful information; entirely fails to answer the question.	Incoherent, meaningless, or impossible to read/follow.	Completely irrelevant. Contains no useful or safe clinical advice.

### Statistical analysis

Statistical analyses were performed using SPSS Statistics version 26.0 (IBM Corp., Armonk, NY, USA). Descriptive statistics were calculated as means and standard deviations (SD) for continuous variables. Inter-rater reliability was assessed using the Intraclass Correlation Coefficient (ICC) with 95% confidence intervals (CI), employing a two-way mixed-effects model for absolute agreement [23]. Internal consistency was evaluated using Cronbach’s alpha coefficient [24]. The Kruskal-Wallis H test was employed to compare performance across the six LLM platforms for each evaluation parameter. Statistical significance was set at *p* < 0.05 for all analyses.

## Results

### Inter-rater reliability and internal consistency

Model-specific reliability coefficients varied significantly. Gemini 2.0 demonstrated the highest consistency (ICC = 0.882, *p* < 0.001), indicating high response stability and structural uniformity that allowed evaluators to score with excellent agreement. Conversely, Microsoft Copilot showed the lowest agreement (ICC = 0.351, *p* = 0.145), suggesting higher intra-model heterogeneity or ambiguous response structures that led to divergent evaluator interpretations. Notably, the ICC for ChatGPT-4o (ICC = 0.659, *p* = 0.062) indicated a trend towards moderate reliability but did not reach the threshold for statistical significance (Table 3).

**Table 3:** Inter-rater reliability and internal consistency by LLM platform

Model	Cronbach’s α	ICC (Single Rater)	ICC (Average)
ChatGPT-4o	0.659	0.492 (*p* = 0.062)	0.659 (*p* = 0.062)
Claude 3.7 Sonnet	0.777	0.635 (*p* = 0.018*)	0.777 (*p* = 0.018*)
Microsoft Copilot	0.520	0.351 (*p* = 0.145)	0.520 (*p* = 0.145)
DeepSeek-V2	0.886	0.795 (*p* = 0.002*)	0.886 (*p* = 0.002*)
Gemini 2.0	0.938	0.882 (*p* < 0.001*)	0.938 (*p* < 0.001*)
Grok-3	0.727	0.571 (*p* = 0.033*)	0.727 (*p* = 0.033*)
Overall	0.709	0.550 (*p* < 0.001*)	0.709 (*p* < 0.001*)

*LLM* Large Language Model, *ICC* Intraclass Correlation Coefficient, α Cronbach’s alpha

* Indicates statistical significance (*p* < 0.05)

### Comparative performance across evaluation parameters

The mean scores for each LLM across all evaluation parameters are summarized in Table 4. Claude 3.7 Sonnet achieved the highest overall mean score (3.79 ± 0.74), demonstrating balanced performance across all metrics. Gemini 2.0 followed closely (3.75 ± 0.58), while Grok-3 ranked third (3.70 ± 0.54). ChatGPT-4o (3.40 ± 0.64), DeepSeek-V2 (3.68 ± 0.46), and Microsoft Copilot (3.29 ± 0.53) demonstrated varying performance levels.

**Table 4:** Comparative performance of LLM platforms across evaluation parameters (Mean ± SD)

Model	Scientific Accuracy	Comprehensiveness	Clarity	Relevance	Total Score
ChatGPT-4o	3.60 ± 0.66	3.40 ± 0.70	3.25 ± 0.79	3.35 ± 0.75	3.40 ± 0.64
Claude 3.7 Sonnet	4.00 ± 0.71	3.45 ± 0.76	3.85 ± 0.82	3.85 ± 0.88	3.79 ± 0.74
Microsoft Copilot	3.45 ± 0.69	2.95 ± 0.55	3.20 ± 0.54	3.55 ± 0.60	3.29 ± 0.53
DeepSeek-V2	3.95 ± 0.69	3.70 ± 0.48	3.50 ± 0.41	3.55 ± 0.64	3.68 ± 0.46
Gemini 2.0	3.90 ± 0.57	3.90 ± 0.66*	3.50 ± 0.58	3.70 ± 0.67	3.75 ± 0.58
Grok-3	4.05 ± 0.50	3.80 ± 0.75	3.50 ± 0.62	3.45 ± 0.69	3.70 ± 0.54
p-value	0.320	0.036*	0.184	0.608	0.300

*LLM* Large Language Model, *SD* Standard Deviation

* Indicates statistical significance (*p* < 0.05)

Statistical analysis using the Kruskal-Wallis H test revealed no statistically significant differences among models for scientific accuracy (*p* = 0.320), clarity (*p* = 0.184), or relevance (*p* = 0.608). However, a statistically significant difference was identified in comprehensiveness (*p* = 0.036). The effect size for this difference, calculated based on the Kruskal-Wallis H statistic, was η² = 0.19, indicating a large effect magnitude among the evaluated models. Post-hoc pairwise comparisons confirmed that Gemini 2.0 (3.90 ± 0.66) provided significantly more comprehensive responses than Microsoft Copilot (2.95 ± 0.55), which frequently offered summarized or superficial information. Notably, while Grok-3 achieved the highest nominal score for scientific accuracy (4.05 ± 0.50), and Claude 3.7 Sonnet excelled in clarity (3.85 ± 0.82) and relevance (3.85 ± 0.88), none of the evaluated models achieved mean scores in the “very good” category (≥ 4.5) for any parameter, indicating room for improvement across all platforms.

## Discussion

This study evaluated the efficacy of six advanced LLM platforms in the specific domain of prosthodontic patient education using a rigorous multi-parameter assessment methodology. The findings led to partial rejection of the null hypothesis: while the evaluated models performed similarly in scientific accuracy, clarity, and relevance, they differed significantly in comprehensiveness—a critical dimension for effective patient education.

The superior comprehensiveness demonstrated by Gemini 2.0 (3.90 ± 0.66) could be attributed to its multimodal architecture and advanced reasoning pathways [25, 26]. These capabilities enable the model to process and synthesize information from diverse data modalities simultaneously, which may facilitate more nuanced integration of complex clinical information compared to purely text-based architectures [4]. In prosthodontics, where patients often require explanations about multi-stage treatments (e.g., implant osseointegration timelines, prosthetic material options, or maintenance protocols), superficial brevity represents a significant limitation. Comprehensive responses that address potential follow-up questions preemptively can reduce patient anxiety and improve informed decision-making [27]. Conversely, Microsoft Copilot exhibited significantly lower comprehensiveness scores (2.95 ± 0.55). This limitation likely stems from its primary optimization as a productivity-focused ‘generative assistant’ integrated with search engine algorithms [28]. Its response strategy appears prioritized toward providing rapid, summary-style information rather than exhaustive clinical explanations [29]. Furthermore, its strict built-in safety constraints and conversational design often lead to brief, generic answers that rapidly defer to professional consultation, thereby omitting necessary preliminary details [30].

Claude 3.7 Sonnet’s achievement of the highest overall score (3.79 ± 0.74) and superior performance in clarity (3.85 ± 0.82) and relevance (3.85 ± 0.88) support claims regarding its Constitutional AI training framework, which prioritizes helpfulness, harmlessness, and coherent dialogue [31]. This makes it particularly suitable for explaining sensitive topics such as denture adaptation challenges, implant failure risks, or aesthetic limitations—areas where balanced, empathetic communication is essential [32].

It is noteworthy that Grok-3 achieved the highest nominal accuracy score (4.05 ± 0.50), although this difference did not reach statistical significance. This suggests that newer, large-parameter models are increasingly capable of minimizing “hallucinations”—the phenomenon where AI systems fabricate medical facts or citations [33]. Nevertheless, it must be strongly emphasized that hallucinations remain an inherent and unpredictable risk across all current platforms. To better understand the clinical implications of these inaccuracies, future research should incorporate a qualitative analysis of the specific factual errors generated during such evaluations. However, the absence of any model achieving ratings in the “very good” category (≥ 4.5) across all parameters reinforces the professional consensus that AI tools should remain adjunctive rather than primary sources of medical information [34].

Our findings align with recent studies evaluating AI performance across various dental and medical specialties. In pediatric dentistry, for instance, chatbots demonstrated accuracy rates between 57% and 78%, yet significantly lacked the nuanced judgment required for clinical decision-making [35]. Similarly, in the field of endodontics, AI models continue to exhibit substantial limitations regarding validity and reliability [36]. Orthodontic literature further confirms that while models excel in general information dissemination, they encounter significant comprehensiveness issues when addressing specific treatment protocols such as clear aligners [37, 38]. Beyond dentistry, broad medical AI research indicates that while LLMs generate scientifically accurate text, they frequently omit secondary clinical details crucial for practical application [19, 39]. A recent evaluation by Sezer and Aydoğdu [40] in dental traumatology noted high consistency in Claude models but reported varying readability metrics. Interestingly, model efficacy also appears to fluctuate significantly depending on the dental specialty being queried. For instance, a recent study by Chatzopoulos et al. in periodontology reported that ChatGPT 4.0 achieved the highest performance metrics, while Google Gemini received the lowest scores in comprehensiveness and scientific accuracy [22]. These periodontal findings contrast directly with our results, where Gemini 2.0 demonstrated superior comprehensiveness and structural consistency. This underscores that LLM capabilities are highly domain-specific and influenced by the distinct terminological and conceptual demands of each specialty. Ultimately, our study extends this broader evidence base specifically to prosthodontics—a domain requiring the explanation of complex mechanical and biological principles to lay audiences [17].

The good inter-rater reliability (ICC = 0.709) observed in this study demonstrates that experienced clinicians can consistently evaluate AI-generated content when provided with structured assessment criteria. This finding supports the feasibility of incorporating expert review processes in future AI integration protocols [41]. The variation in model-specific reliability coefficients reveals important differences in response stability. The excellent inter-rater agreement for Gemini 2.0 (ICC = 0.882) suggests high structural stability and predictable comprehensiveness in its outputs, allowing clinicians to evaluate it consistently. In contrast, the poor agreement for Microsoft Copilot (ICC = 0.351) and its significantly less comprehensive responses highlight the impact of its specific interface. Unlike purely generative models, Copilot often synthesizes real-time web search results; this dynamic retrieval process compromises clinical consistency, producing highly variable, summary-style responses [28, 29] that evaluators interpreted differently regarding clinical depth. These platform-specific consistencies are highly relevant for future institutional adoption decisions.

From a clinical perspective, these findings suggest that LLM chatbots may serve useful supplementary roles in patient education workflows, particularly for providing preliminary information, addressing frequently asked questions, or offering after-hours access to basic dental health information. However, the moderate performance levels observed underscore that these tools cannot replace professional consultation, personalized treatment planning, or clinical judgment [42].

### Limitations

Several limitations should be acknowledged. First, this study utilized a limited set of ten questions that, while representative of common clinical scenarios, does not encompass the full breadth of prosthodontic practice. Additionally, while this sample size was robustly powered (87%) to detect large effect sizes, this descriptive benchmarking design may be underpowered to identify very subtle clinical differences in parameters where model performance was highly homogeneous, such as scientific accuracy and clarity. Second, the evaluation was conducted in Turkish. While LLMs are highly multilingual, they are predominantly trained on massive English datasets. Consequently, the interpretation and cognitive processing of highly specific Turkish prosthodontic terminologies might introduce subtle semantic variations, potentially influencing the models’ performance compared to English-language clinical outputs. Third, all assessments were conducted at a single time point; LLM capabilities evolve rapidly, and performance may change substantially within months of publication [18, 19]. Fourth, the study employed prosthodontic specialists as evaluators; patient perspectives on clarity and utility might differ from expert assessments. Therefore, we strongly recommend that future studies include laymen to evaluate the ‘Clarity’ parameter, as the perception of intelligibility can vary drastically between dental professionals and the general public. Finally, the digital benchmarking methodology does not capture real-world clinical contexts where follow-up questions, visual aids, and personalized communication enhance understanding.

## Conclusion

In conclusion, contemporary Large Language Models demonstrate commendable scientific accuracy and clarity when addressing prosthodontic patient inquiries, highlighting their potential as supplementary educational tools. However, there remains substantial room for improvement, particularly in providing comprehensive, detailed information that anticipates patient follow-up questions and outlines specific clinical alternatives. Given the unprecedented pace of AI development and the frequent architectural updates to these platforms, the findings of this study represent a specific temporal benchmark; thus, continuous, rigorously designed evaluations will be essential. Ultimately, rather than replacing professional consultation, the evolving role of AI in dental practice is poised to become a powerful, interactive adjunct—one that enhances patient health literacy, streamlines clinical workflows, and fundamentally reinforces the patient-clinician relationship.

## Supplementary Information

Supplementary Material 1.

## Data Availability

The datasets generated and analyzed during the current study are available from the corresponding author upon reasonable request.

## Abbreviations

AI: Artificial Intelligence
LLMs: Large Language Models
ICC: Intraclass Correlation Coefficient
CI: Confidence Interval
SD: Standard Deviation
STROBE: Strengthening the Reporting of Observational Studies in Epidemiology
MoE: Mixture-of-Experts
SPSS: Statistical Package for the Social Sciences
