# Supplementary material for: Comparative efficacy of six contemporary large language models in prosthodontic patient education: a multi-parameter blinded assessment
Source: BMC Oral Health. 2026 May 27;26:1442. doi: 10.1186/s12903-026-08705-9 (PMC13474867; doi:10.1186/s12903-026-08705-9)
Supplement: Supplementary file 1 [file 12903_2026_8705_MOESM1_ESM.docx]

**Additional file 1: Standardized Patient Questions**

The following standardized questions were developed for this study to simulate common prosthodontic patient inquiries. These questions were posed to each Large Language Model (LLM) in Turkish.

**Question 1:** My prosthesis is causing sore spots in my mouth. What should I do?

**Question 2:** Should I remove my dentures at night?

**Question 3:** Is it safe to have a Magnetic Resonance Imaging (MRI) scan with dental implants?

**Question 4:** What causes the loosening of fixed prostheses (crowns/bridges)?

**Question 5:** How often and how should I clean my dental prosthesis?

**Question 6:** How long do I need to wait after the implant surgery before the prosthesis can be made?

**Question 7:** What is a precision attachment prosthesis? How does it differ from a traditional partial denture?

**Question 8:** What are the differences between domestic and imported dental implants?

**Question 9:** Do dental implants last a lifetime?

**Question 10:** I clench my teeth at night (bruxism), what should I do?

**Türkçe Soru Seti**

**Soru 1:** Protezlerim ağzımda vuruk yapıyor. Ne yapmalıyım?

**Soru 2:** Takma dişlerimi gece çıkarmalı mıyım?

**Soru 3:** İmplantlar MR çekimine engel midir?

**Soru 4:** Sabit protez gevşemesi neden olur?

**Soru 5:** Protez temizliği ne sıklıkla ve nasıl yapılmalı?

**Soru 6:** İmplant cerrahisi uygulaması sonrası protez yapılması için ne kadar süre beklenmelidir?

**Soru 7:** Hassas tutuculu protez nedir? Geleneksel protezden farkı ne?

**Soru 8:** Yerli implantla yabancı implantın farkları nedir?

**Soru 9:** İmplantlar ömürlük müdür?

**Soru 10:** Gece dişlerimi sıkıyorum, ne yapmalıyım?
